# A Medipix quantum area detector allows rotation electron diffraction data collection from submicrometre three-dimensional protein crystals

**DOI:** 10.1107/S0907444913009700

**Published:** 2013-06-15

**Authors:** Igor Nederlof, Eric van Genderen, Yao-Wang Li, Jan Pieter Abrahams

**Affiliations:** a Biophysical Structural Chemistry, Leiden University, Einsteinweg 55, 2333 CC Leiden, The Netherlands

**Keywords:** electron diffraction, electron microscopy, Medipix2, *MOSFLM*, nanocrystals

## Abstract

When protein crystals are submicrometre-sized, X-ray radiation damage precludes conventional diffraction data collection. For crystals that are of the order of 100 nm in size, at best only single-shot diffraction patterns can be collected and rotation data collection has not been possible, irrespective of the diffraction technique used. Here, it is shown that at a very low electron dose (at most 0.1 e^−^ Å^−2^), a Medipix2 quantum area detector is sufficiently sensitive to allow the collection of a 30-frame rotation series of 200 keV electron-diffraction data from a single ∼100 nm thick protein crystal. A highly parallel 200 keV electron beam (λ = 0.025 Å) allowed observation of the curvature of the Ewald sphere at low resolution, indicating a combined mosaic spread/beam divergence of at most 0.4°. This result shows that volumes of crystal with low mosaicity can be pinpointed in electron diffraction. It is also shown that strategies and data-analysis software (*MOSFLM* and *SCALA*) from X-ray protein crystallography can be used in principle for analysing electron-diffraction data from three-dimensional nanocrystals of proteins.

## Abstract 2

An ultrasensitive Medipix2 detector allowed the collection of rotation electron-diffraction data from single three-dimensional protein nanocrystals for the first time. The data could be analysed using the standard X-ray crystallography programs *MOSFLM* and *SCALA*.

## 1. Introduction

Protein crystallography is a major justification for large-scale X-ray facilities such as synchrotrons and free-electron lasers. However, three-dimensional protein crystals that are smaller than about 0.5 µm are too small for standard X-ray crystallography, although XFEL sources are expanding the method towards smaller crystals (Chapman *et al.*, 2011 ▶). This is a serious bottleneck, as about 30% of proteins that crystallize do not grow crystals of sufficient size or quality for X-ray structure determination (Rupp, 2004 ▶). In particular, membrane proteins and large (dynamic) protein–nucleic acid complexes fail to grow into crystals of sufficient size. Structural information on these important drug targets is therefore severely lacking.

Electrons are less damaging to proteins than X-rays by several orders of magnitude per elastically diffracted quantum (Henderson, 1995 ▶). This property of electrons explains the successes of two-dimensional electron crystallography. For instance, 45 Å thick submicrometre patches of two-dimensional bacterio­rhodopsin crystals yielded images with a resolution of 2.8 Å (Baldwin *et al.*, 1988 ▶). Three-dimensional crystals with an equivalent volume would measure approximately 150 × 150 × 150 nm. Recent data demonstrate that useful high-resolution electron diffraction data (up to 2.5 Å resolution) can be obtained from nanosized three-dimensional protein crystals, where synchrotron X-rays fail (Jiang *et al.*, 2009 ▶).

However, only single diffraction shots could be collected. Collection of rotation data from protein nanocrystals was not possible because the signal-to-noise ratio and dynamic range of CCD detectors and image plates was insufficient. Very recently, direct electron detectors have become available which have a better signal-to-noise ratio and which may be better suited. These new detectors are very expensive and are probably not sufficiently radiation-hard to be routinely exposed to the direct electron beam.

Electrons can also be detected by quantum area detectors such as the Medipix2 (Llopart Cudié *et al.*, 2002 ▶; Faruqi & Henderson, 2007 ▶). The Medipix2 detector is more radiation-hard than other direct electron detectors such as the Falcon because its read-out electronic circuitry (which is sensitive to radiation damage and interference) is shielded by a semiconductor sensor layer, to which it is bump-bonded (Llopart Cudié *et al.*, 2002 ▶). The detector has a very high signal-to-noise ratio because each pixel has its own readout electronics that measures the hole-charges that are produced by an incident electron hitting the sensor layer within 10^−5^ s. If the integrated energy after amplification is above a set threshold corresponding to the energy of a 200 keV electron the incident quantum is counted as a ‘hit’. Thus, the Medipix2 chip only counts 200 keV electrons and, unlike many other detectors, is blind to soft X-rays of lower energy that are also produced in great abundance inside any electron microscope. In this fashion its noise is almost exclusively determined by the counting statistics of the electrons. This gives a significant improvement over conventional CCD cameras in electron microscopes (Faruqi & McMullan, 2011 ▶; Georgieva *et al.*, 2011 ▶).

We recently showed that a Medipix2 detector with a 500 µm sensor layer can detect 200 keV electrons with a signal-to-noise ratio that is at least an order of magnitude better than that of image plate (Georgieva *et al.*, 2011 ▶). The Medipix detector is highly sensitive at low count rates, allowing accurate measurement of the high-resolution terms. In addition, with a 500 µm sensor layer it is sufficiently radiation-hard to routinely be exposed to a direct beam of 200 keV electrons. It also has a high dynamic range, allowing accurate measurement of the intense highly peaked dose that it receives in the low-resolution diffraction spots. At 200 keV, the point spread of the detector is increased, but if the point spread is not much higher than the spread of the Bragg spots this would not matter for collecting diffraction data.

In view of these desirable characteristics of the Medipix2 detector, we investigated whether it would be possible to reduce the electron dose per diffraction frame by an order of magnitude and still measure high-resolution diffraction data, as this would allow the collection of rotation diffraction data from single protein crystals for the first time.

## 2. Materials and methods

### 2.1. Crystallization

**Figure 1:**
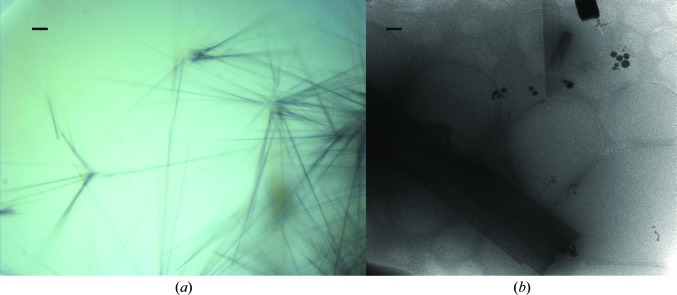
Lysozyme nanocrystals in a crystallization drop (*a*) and an electron micrograph of vitrified crystals (*b*). Scale bars correspond to 10 µm (*a*) and 500 nm (*b*).

Crystallization experiments were carried out using the sitting-drop vapour-diffusion technique in Innovadyne SD-2 plates. The *Rock Maker* software (Formulatrix) was used to design the experiments. A Genesis (Tecan) robot was used to dispense the screening solutions in the reservoirs of MRC2 plates (Swissci). An Oryx 6 (Douglas Instruments) crystallization robot was used to transfer 500 nl reservoir solution and 500 nl protein solution into sitting-drop wells. Plates were stored at 291 K and imaged using a Rock Imager automated imaging system (Formulatrix). Lysozyme (8 mg ml^−1^) formed needle-shaped crystals after 48 h when mixed in a 1:1 ratio with well solution consisting of 0.1 *M* sodium acetate pH 3.8, 1.0 *M* potassium nitrate (Fig. 1 ▶).

### 2.2. Vitrification

Protein crystals were vitrified using a Vitrobot (FEI). 3 µl well solution was mixed with the drop containing nanocrystals and transferred to a 3 mm glow-discharged holey carbon grid (AGAR). Excess liquid was blotted away (blot time 3 s, blot force 5) and the sample was plunge-frozen in liquid ethane cooled by liquid nitrogen.

### 2.3. Rotation electron diffraction

Diffraction data were collected at 200 keV on a CM200FEG (Philips) transmission electron microscope at the National Center for High Resolution Electron Microscopy in Delft. Samples were cooled to 93 K in liquid nitrogen in an in-house-modified cryo-transfer holder (Gatan). Diffraction patterns were collected using a Medipix2 detector. We created a narrow highly parallel electron beam with limited intensity by using a small (10 µm) condenser aperture and spot size 11 (which controls the first condensor lens). The beam was hardly convergent, as indicated by the data analysis described below (<0.4°). An automated compu-stage allowed crystal alignment and rotation.

### 2.4. Measuring electrons using a Medipix2 detector

**Figure 2:**
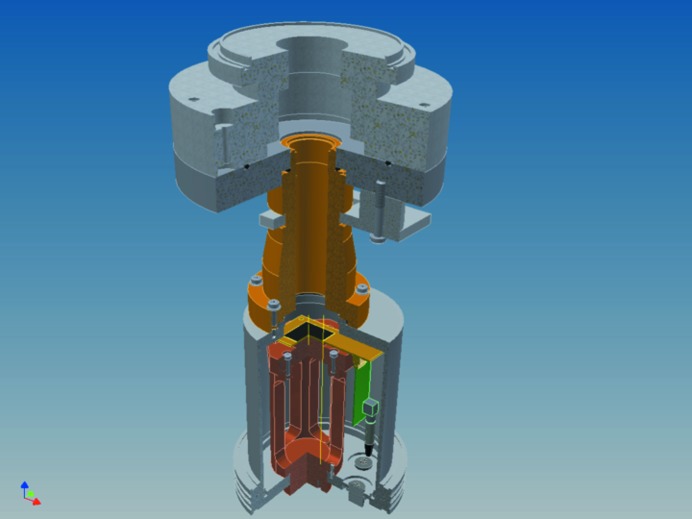
Cutout view of the Medipix2 camera assembly. Electrons enter the tube from the top and hit the detector (black square) on the Nikhef assembly board (San Segundo Bello *et al.*, 2003 ▶), which is connected to the computer through the USB read-out electronics and a vacuum connector.

Diffraction patterns were collected with a CMOS Medipix2 detector mounted on a Nikhef carrier board (Fig. 2 ▶) and the data were transferred to a Windows PC using USB1.1 read-out electronics (Vykydal *et al.*, 2006 ▶). Four abutting Medipix2 ASICs, each with 256 × 256 pixels and a pixel size of 55 µm, make a Medipix2 Quad. The Quad was covered by a single custom-made 500 µm semiconductor sensor chip. The distance between two neighbouring single Medipix2 ASICs is approximately 250 µm, so the edge pixels are about 125 µm wide. Therefore, they will capture more signal than the other pixels, resulting in a bright cross that quarters the raw images. This cross can be corrected for, as discussed below.

The detector has a large dynamic range, where each pixel consists of a separate 14-bit pseudo-random counter. The test circuitry and four-bit trimming system are able to compensate for most fabrication variations and therefore the overall global threshold has a variation of 95 eV. The electronics noise can account for another 110 eV. These two values combined result in a dynamic range of no lower then 900 eV between channels. In silicon ∼3.6 eV is required to produce one electron–hole pair; therefore, a 200 keV electron can produce ∼55 000 pairs. This means that the dynamic range of the electronic noise accounts for less than 1.6% of the total deposited energy per electron incident and even less when there is more than one electron incident per clock cycle (McMullan *et al.*, 2007 ▶; Plackett *et al.*, 2010 ▶).

The thickness of the sensor layer (500 µm) is larger than for stock Medipix2 chips (300 µm). We selected this larger thickness to prevent the 200 keV electrons from penetrating through the sensor layer and damaging the underlying electronics. Monte Carlo simulations (300 µm Si sensor layer; 120, 200 and 300 keV) predicting such events have been described previously (Faruqi & Henderson, 2007 ▶; McMullan *et al.*, 2009 ▶; Turecek *et al.*, 2011 ▶). Most importantly, they also describe events where these higher energy electrons scatter in the silicon layer to neighbouring pixels. As long as this scattering stays close to the boundaries of the Bragg spots, this would give no major point-spread problems.

There is a need to set the threshold settings such that the gain of the detector is close to or slightly lower than one. Setting the threshold too low will result in multiple pixels recording a single high-energy electron hit; setting the threshold too high will not count electrons or (at sub-optimally high thresholds) will only count electrons that deposit all of their energy into a single pixel. It is important that the threshold is chosen in such a way that when the electron partly scatters to a neighbouring pixel it still is counted. The chance of a single high-energy electron scattering in the sensor layer to neighbouring pixels increases with higher energies (Faruqi & McMullan, 2011 ▶). To obtain a higher electron-counting gain, a compromise threshold level was chosen where lower energy particles would also be counted in each pixel. This, however, is not of much influence in electron-diffraction studies with the Medipix, since most other particles (X-rays) will stay well below an equivalent 100 keV threshold setting. This would be an undesirable setting in any case, because it would enable double counting in neighbouring pixels for a single-electron hit. To minimize radiation damage the experiment was conducted under such low-dose conditions that the chance of a two-electron hit within the same pixel within a clock cycle of the camera is almost non-existent (*e.g.* less then 0.1%). We validated our gain estimate with *MOSFLM*. The spot-profile statistics predicted a gain of 0.816 counts per electron. However, since the measurement was performed at very low dose conditions (the average count per pixel per frame is around five) it is no longer possible to estimate the gain by fitting a Gaussian (the *MOSFLM* method) instead of a Poisson distribution. Therefore, the true gain must be higher than 0.816 but not higher than 1.00.

**Figure 3:**
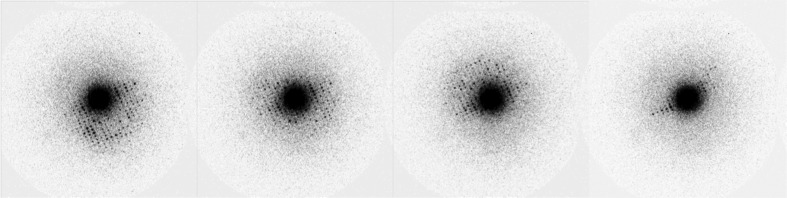
Four diffraction patterns from a series of 23 consecutiveframes (0.05° per frame, 0.083° between adjacent images; see text for details) collected at 200 keV with a Medipix2 Quad detector (512 × 512 pixels). The crystal was orthorhombic lysozyme, maximum resolution ∼1.8 Å. The frame on the far right is after 30 s of data collection and shows the degradation in signal arising from beam damage. Each frame was exposed to between 0.05 and 0.1 e^−^ Å^2^ per second.

The vacuum pod in which the Medipix2 was mounted on the on-axis port of the CM-200 FEG electron microscope is shown in Fig. 3 ▶. It houses both the carrier and the USB1.1 readout board. Damage to the electronics of these boards by the direct electron beam was prevented by the small window of the pod, which only allowed illumination of about 80% of the total area of the Medipix2. Out-gassing of the electronics at 1 × 10^−6^ Pa vacuum did not prove to be a problem for either the electronics or the TEM. The bias voltage on the Medipix2 was set to 100 V to be able to count the holes after the electron incidents (Georgieva *et al.*, 2011 ▶). The four chips are set to nonparallel readout by the *Pixelman* v.2.0 software (Turecek *et al.*, 2011 ▶) on a PC with Windows 7 in Java mode. The Medipix2 Quad was calibrated using the standard tools that are included in *Pixelman*.

(i) DACs were scanned to determine the noise edge of the different ASICs.
(ii) Threshold equalization was performed to remove small standard discrepancies between individual pixels (3 bits). This procedure adjusts each pixel so that its threshold is as close as possible to the average and was performed with the standard settings supplied with the *Pixelman* package.
(iii) Each lower threshold (THL) was set to a value close to the noise edge in order to acquire as much signal as possible from the incident electrons.
(iv) Flat-field images obtained by illumination with a uniform beam allowed equalization of the four chips.

### 2.5. Preparing diffraction patterns for data processing

The Medipix2 images need some pre-processing before they can be read into *MOSFLM*. Three problems needed to be addressed: (i) the cross needed to be removed, (ii) dead pixels needed to be corrected and (iii) the image needed to be centred, as there was a substantial drift of the direct beam. With this aim, we wrote a program that solved these issues as described below.

#### 2.5.1. Removing the cross

A bright cross appears in the raw Medipix2 Quad images as it is a tiled assembly of four single Medipix2 ASICs. The edge pixels of each single Medipix2 ASIC that abuts another in the Quad assembly are larger than the other pixels (they are 125 µm wide rather than 55 µm). Therefore, the pixels at the interface between single Medipix2 ASICs capture more electrons. This results in spatial distortion and non-uniformity of the measured signal. In order to correct for the spatial distortion, our program shifts pixels with an *x* value smaller than 256 by one pixel to the left and shifts pixels with a value of 256 or higher by one pixel to the right. It applies vertical shifts in a similar fashion. This procedure duplicates the pixels adjacent to the horizontal and vertical bisecting lines (and quadruples the four centre pixels). Their value was divided by 2.3 (or 5.3 for the quadrupled centre pixels) to correct the increased measured intensities of these pixels.

#### 2.5.2. Correcting bad pixels

Despite tuning the pixels of the Medipix2 detector, it can still have dead pixels (or bright pixels). Since the positions of dead pixels are fixed and their values do not follow a Poisson distribution over time, it is possible to identify these dead pixels. For each pixel position, we calculated its variance over a range of images and compared this variance with the variances at the other positions. ‘Good’ pixels will have a much higher variance than ‘bad’ pixels since the values of the latter tend to be relatively constant and largely independent of the signal, although an occasional bad pixel may be hypervariable with a large variance. If the variance value was below a set threshold the pixel was labelled as ‘bad’. After having identified the bad pixels, every image was corrected. For every bad pixel in every image, its value will be replaced by the mean value of the good pixels that surround it. If a bad pixel had no good neighbours the procedure was iterated after reclassification of the pixels.

#### 2.5.3. Centring the images

The position of the beam centre shifted as a function of the tilt of the sample holder. The shift was not huge (15 pixels maximally), but was sufficiently large to interfere with data processing. Images were centred by calculating their cross-correlations with a two-dimensional Gaussian function peaking at pixel (256, 256) and applying the calculated shift. After centring the images, they were written to disk in the *CCP*4 .pck format (Abrahams, 1993 ▶) as 1200 × 1200 pixel images so that they could be processed as small MAR images by *MOSFLM* (Leslie & Powell, 2007 ▶).

## 3. Results

### 3.1. Collecting diffraction data

Prior to installing the Medipix2 detector in the electron microscope, we collected diffraction data on an image plate, film and CCD, but with these detectors we were never able to collect multiple diffraction patterns from single protein crystals to high resolution because of beam damage (Jiang *et al.*, 2009 ▶). The Medipix2 detector has an efficiency at low count rates that is at least an order of magnitude higher than that of an image plate (Faruqi & McMullan, 2011 ▶; Georgieva *et al.*, 2011 ▶). This meant that for the first time we could collect rotation data over multiple images without severe beam damage.

Unfortunately, the hardware prevented us from interfacing the detector with the electron illumination and the rotation of the sample holder. This meant in practice that we had to start rotating the crystal slowly, switch on the beam and start collecting images until the crystal had sustained too much damage. Because transferring a single Medipix2 frame into the computer takes 0.7 s (we were using a USB1 interface), this meant that we could only collect rotation data with angular gaps between adjacent images. By using fine-ϕ slicing, we reduced the systematic errors that were introduced by this unfortunate but in the circumstances inevitable experimental flaw.

**Figure 4:**
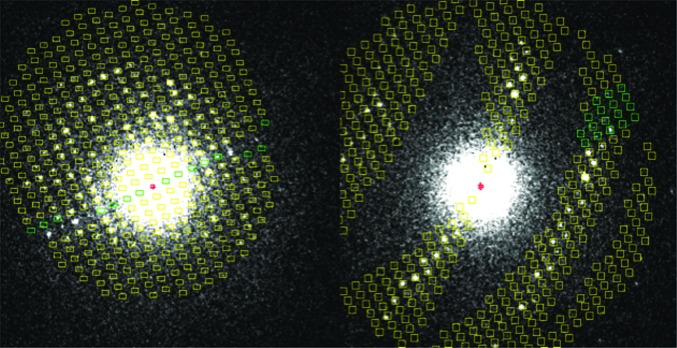
Overlay of predicted spot positions (resolution threshold 2 Å) with observed diffraction patterns of lysozyme nanocrystals, indicating that the *a* axis could be close to 125 Å, while the *b* and *c* axes are about 34.2 and 25.5 Å, respectively. The images are screen shots of *MOSFLM* runs.

We collected rotation data from many crystals, improving the data-collection strategies and sample handling iteratively. Here, we describe representative results using two rotation data sets from the same single crystal but collected at different positions using a fresh part of the crystal. We rotated in the positive direction for the first wedge of data and in the negative direction for the second wedge. Both wedges started at the same goniometer setting, but processing revealed their orientations to be about 1.5° apart. The first wedge was measured with a rotation speed of 0.083° per image (0.05° s^−1^), reading out an image every second. This resulted in a data set in which each image had recorded about 0.050° of rotation data, with a gap of 0.033° until the next image. For the second data set the crystal was rotated at a speed of 0.2° s^−1^, reading out an image every second. This resulted in a data set with 0.15° per image and gaps between images of 0.11°. Both data sets had diffraction spots to a resolution of 1.8 Å in the early images (see Fig. 4 ▶).

**Figure 5:**
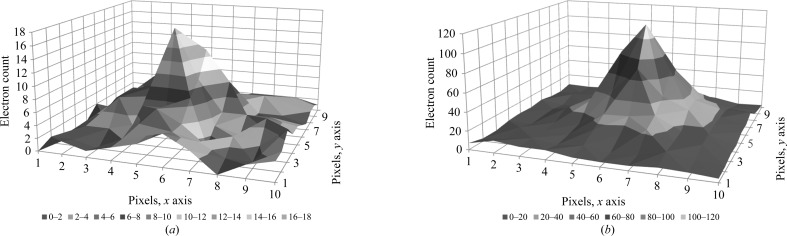
Two typical spot profiles in a 10 × 10 pixel frame. (*a*) A low-intensity spot profile at 2.0 Å resolution with a maximum of 15–20 electrons. (*b*) A high-intensity spot profile with a maximum of 100–120 electrons close to the central beam.

The diffraction spots were analysed and the profiles of two single spots are shown in Fig. 5 ▶. Low-intensity spots (15–20 electrons in the peak) that are far from the central beam clearly show up above the noise (Fig. 5 ▶ *a*). These spots still show a significant level over the surrounding background pixels, which on average count 2–­3 electrons. For comparison, a bright spot with a maximum intensity of 100–120 electrons per pixel is shown in Fig. 5 ▶(*b*).

### 3.2. Processing the diffraction data with *MOSFLM*

Several essential parameters had to be extracted from the diffraction patterns: the angle of the rotation axis relative to the detector coordinate frame, the beam centre, the beam divergence, the unit-cell parameters and the orientation of the crystal. The determination of each of these parameters will be discussed in more detail below.

#### 3.2.1. Beam centre

We observed the beam centre to shift considerably (about 15 pixels) between the starting and ending rotation angles. Since we did not use a backstop, this shift could readily be corrected after data collection by shifting back the images accordingly (as described in §[Sec sec2]2). At the time of data collection it was unclear what the cause of the beam shift was, but it later transpired that a screw of the EM stage close to the crystals had become slightly magnetized.

#### 3.2.2. Angle of the rotation axis

As the electrons spiral their way through the magnetic lenses of the microscope, the rotation axis that is observed on the detector is not necessarily an orthogonal projection of the physical rotation axis. It needs to be calibrated for every camera distance. Careful analysis of the diffraction patterns using the human eye, a ruler and a protractor indicated that at a virtual camera length of 700 mm and an electron energy of 200 kV the angle between the rotation axis and the *x* direction of the detector was around 115°. We could refine this initial estimate to 116.5° by overlaying predicted diffraction patterns on observed diffraction patterns once we had good estimates of the other parameters. When rotating in the negative direction, the rotation axis was redefined to be −63.5°.

#### 3.2.3. Unit-cell parameters

Using our knowledge of the virtual crystal-to-film distance and the wavelength (which is 0.0251 Å for electrons at 200 keV energy), we interactively estimated the two spacings in the plane of the detector using the ‘measure’ facility in *MOSFLM*. These parameters (34.2 and 25.5 Å with an angle of 90°) were consistently found in both rotation ranges. No low-index spacings of the third axis were present in the images, and its magnitude could only be estimated from the location of the lunes in the second data set. A reasonable, but by no means perfect, overlay between observed and predicted spot positions could be obtained with a unit cell of *a* = 125, *b* = 34.2, *c* = 25.5 Å, with all cell angles 90° (Fig. 4 ▶). In the past we found orthorhombic nanocrystals of lysozyme to have *P*2_1_2_1_2_1_ symmetry, with a unit cell of around *a* = 31.5, *b* = 52.5, *c* = 89 Å (Jiang *et al.*, 2009 ▶). However, we could not index the rotation data with the latter unit-cell parameters.

#### 3.2.4. Determining the orientation of the crystal

Owing to radiation damage, we could only measure small wedges of data. Auto-indexing routines could not cope with the data. Therefore, we indexed by eye and let *MOSFLM* refine the orientation using post-refinement, which, despite the angular gaps between images, managed to improve the fit between the observed and predicted patterns. The wedges contained sufficient data for orientation refinement, but did not allow refinement of the unit-cell parameters.

#### 3.2.5. Beam divergence

Once we had satisfactory predictions of the diffraction patterns, we adjusted the beam divergence (or mosaic spread; the two phenomena cannot easily be disentangled) in *MOSFLM* so that all spots were covered by the predicted pattern. The combined beam divergence/mosaic spread was about 0.3–0.4°.

**Figure 6:**
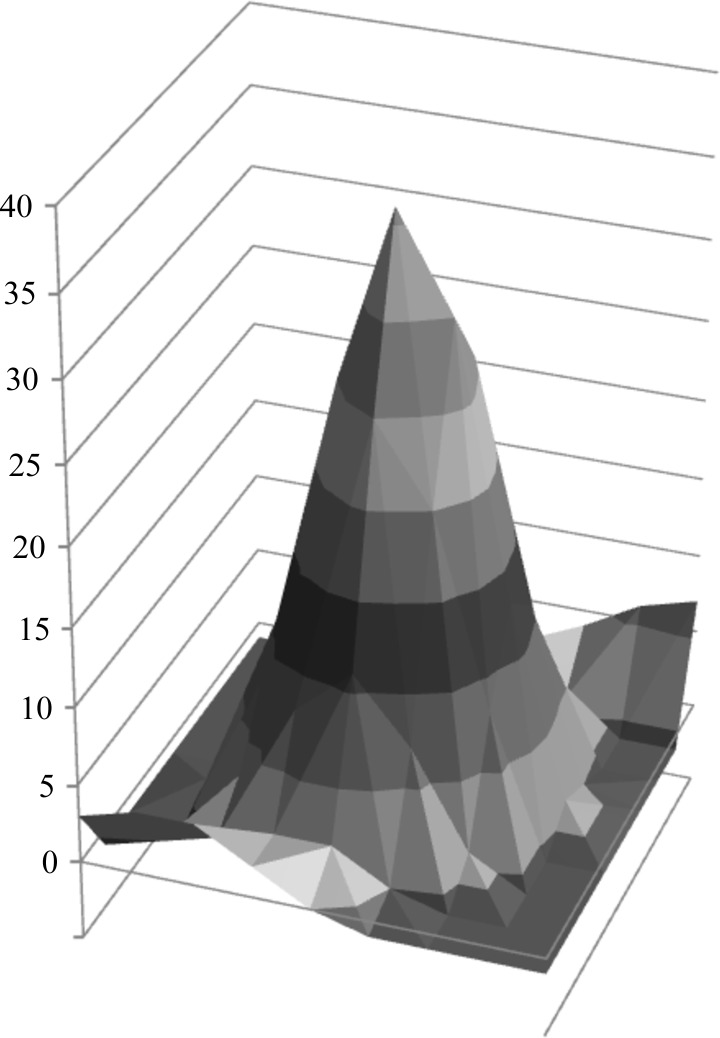
Spot profile from *MOSFLM* for the first data set. The second data set had a similar spot profile (not shown).

Typical profiles of the spots are shown in Fig. 6 ▶. There were a number of bad spots, mainly because of a high gradient in the background, but by setting the maximum gradient to five (instead of the default value of three) all spots were accepted. One likely cause of the high background gradient for some of the spots was the very intense zero-order reflection. Removing this reflection with a backstop was not possible without also removing all of the reflections with a resolution lower than about 5 Å. The reason for this is the low scattering angle and the position of the backstop in the CM-200 microscope. For protein crystallographic data collection the position of the backstop should have been much closer to the detector, but hardware restrictions prevented us from moving the backstop.

**Figure 7:**
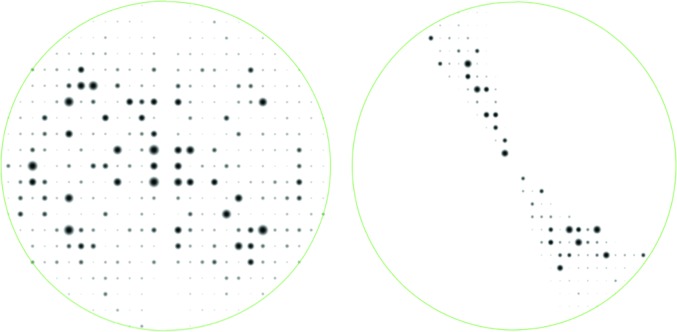
Laue zones at *h* = 0 of the scaled data as displayed by *VIEWHKL*. Note that the second data set (right pattern) does not have a complete *h* = 0 Laue zone.

Scaling the data was problematic in view of the high partiality, the small wedge size, the scarcity of data and the angular gaps between the frames. For this purpose, we used the *CCP*4 program *SCALA* (Evans, 2006 ▶). The statistics were poor because we had to scale the partials using their calculated partiality and because of the large missing gaps of data between adjacent images. Fig. 7 ▶ shows the merged and scaled *h* = 0 zones of both data sets. Note that we processed all of the data as *P*1 with anomalous signal. We did so in order to identify potential dynamic scattering, which results in a breakdown of point-group symmetry in the diffraction patterns (Abrahams, 2010 ▶). The symmetries shown in Fig. 7 ▶ are therefore intrinsic to the diffraction experiment and not imposed by symmetry averaging of corresponding reflections.

## 4. Discussion and conclusion

Three-dimensional nanocrystals of lysozyme have successfully been grown, vitrified and transferred to an electron microscope at liquid-nitrogen temperature. Rotation electron-diffraction data were obtained at multiple positions of a single submicrometre crystal to resolutions better than 2 Å. For the first time, we could collect multiple frames from protein nanocrystals. This was made possible by the much higher sensitivity and signal-to-noise ratio of the Medipix2 detector, combined with its radiation hardness at 200 keV.

By using a parallel electron beam, we could observe the curvature of the Ewald sphere in electron-diffraction patterns of protein crystals for the first time. At 200 keV, electrons have a wavelength only about 0.025 Å, causing the Ewald sphere to be extremely flat at low resolution. Yet the data show that the curvature is still significant.

When the curvature of the Ewald sphere cannot be ignored, both members of a Friedel pair cannot simultaneously be in full diffraction. Geometric considerations imply that the rotation angle between the two reflections of a Friedel pair is always at least their diffraction angle. Consider, for instance, a reflection at 5 Å resolution and assume that it is optimally aligned. At λ = 0.025 Å the crystal would need to be rotated by almost sin^−1^(0.025/10) = ∼0.3° in order to move its Friedel mate into full diffraction. The fact that we can clearly see deviations in intensity between Friedel mates even at 5 Å resolution (Fig. 4 ▶) confirms the low beam divergence and mosaic spread suggested by the data processing. Clearly, the rocking curve of the crystal (with a maximal width of less than 0.4°) is sufficiently peaked to allow the observation of these fine angular differences in orientation. This implies that it should be possible to pinpoint areas of low mosaicity in the crystals. This may have an advantage if the crystalline order varies within a single nanocrystal, in which case its best parts can be selected.

These results are encouraging enough to consider fixing the technical problems that prevent optimal data collection. The lack of a scheduling interface between the detector and the goniometer that controls the crystal orientation, combined with the relatively slow readout of the Medipix2 Quad (0.7 s), is an evident cause of many of these problems. As the rotation of the sample could not be interrupted during the readout time, there were angular gaps between subsequent frames. We anticipate fixing this problem by employing a novel Medipix2 detector on a Relaxd read-out board (Visser *et al.*, 2011 ▶) with improved readout time (up to 200 frames per second) that can also read data while collecting the next frame. This would significantly decrease the illumination time and subsequent beam damage. We plan to mount the detector on a Titan Krios machine, so data collection will be improved owing to the presence of an autoloader, a programmable goniometer and a programmable beam blanker. For small-molecule crystals, it has been reported that the collection of data in STEM diffraction mode further reduces beam damage (Kolb *et al.*, 2011 ▶), so we plan to incorporate the Medipix2 detector in a microscope that has this option.

In view of the suboptimal data collection, we were happy being able to integrate the data with one of the standard programs for processing X-ray data: *MOSFLM*. However, we cannot exclude the possibility that the results that we report here do not reflect the true crystal structure. We noticed unexpected correlations between parameters. For instance, rotation of the crystal around ϕ was accompanied by an apparent rotation (as suggested by post-refinement) around Φ*X*, which is a rotation of the crystal around the beam. This may perhaps be caused by eucentric height variation of the crystal as it is being rotated. Since the electrons are focused spirally, rotation of the image or diffraction pattern with height is expected. These and other correlations, which were independent of the unit-cell or crystal-orientation parameters, may indicate that the indexing solution that we present here is wrong. However, we think that these correlations are caused at least in part by distortions specific to electron diffraction. These exist and we have unequivocally identified at least one. For instance, the correlation of the beam shift with rotation of the crystal, which we have observed (and corrected for), would never be encountered in X-ray diffraction.

Together with beam damage, the angular gaps between adjacent images may explain the observed breakdown in point symmetry observed in the *h* = 0 Laue zones (Fig. 7 ▶). An alternative explanation may be found in the effects of dynamic scattering, which also causes such a breakdown in symmetry (Abrahams, 2010 ▶). With improved hardware we anticipate being able to distinguish between these effects.

We have demonstrated that it is possible, at least in principle, to collect rotation electron-diffraction data from protein nanocrystals. This prompts the question concerning the usefulness of such data sets in solving protein crystal structures. Problems with dynamic scattering have been anticipated, yet these did not prevent structure solution of the aquareovirus to 3.3 Å resolution by single-particle analysis (Zhang *et al.*, 2010 ▶) using crystals that also had a size of approximately 100 nm, just like those analyzed here. If at higher resolution the effects of dynamical scattering can no longer be ignored, they can be modelled by the multi-slice approach (Jansen *et al.*, 1998 ▶). This is common practice in electron crystallography of small molecules. Furthermore, there are theoretical considerations that imply that dynamical scattering may yield phase information (Abrahams, 2010 ▶) and a limited amount of such dynamical scatter may therefore even be beneficial.

Electron area detectors such as Medipix that allow the distinction of the signal of high-energy electrons from that of other types of radiation are likely to have a major impact on structural biology. Our results imply that protein crystallo­graphy also stands to benefit from these technical advances.
